# Alpha-Synuclein Induces Lysosomal Rupture and Cathepsin Dependent Reactive Oxygen Species Following Endocytosis

**DOI:** 10.1371/journal.pone.0062143

**Published:** 2013-04-25

**Authors:** David Freeman, Rudy Cedillos, Samantha Choyke, Zana Lukic, Kathleen McGuire, Shauna Marvin, Andrew M. Burrage, Stacey Sudholt, Ajay Rana, Christopher O'Connor, Christopher M. Wiethoff, Edward M. Campbell

**Affiliations:** 1 Integrated Cell Biology, Stritch School of Medicine, Loyola University Chicago, Maywood, Illinois, United States of America; 2 Department of Microbiology and Immunology, Stritch School of Medicine, Loyola University Chicago, Maywood, Illinois, United States of America; 3 Department of Pharmacology and Experimental Therapeutics, Stritch School of Medicine, Loyola University Chicago, Maywood, Illinois, United States of America; 4 Missouri School of Medicine, Columbia, Missouri, United States of America; 5 Department of Biological Sciences, North Central College, Naperville, Illinois, United States of America; Hertie Institute for Clinical Brain Research and German Center for Neurodegenerative Diseases, Germany

## Abstract

α-synuclein dysregulation is a critical aspect of Parkinson's disease pathology. Recent studies have observed that α-synuclein aggregates are cytotoxic to cells in culture and that this toxicity can be spread between cells. However, the molecular mechanisms governing this cytotoxicity and spread are poorly characterized. Recent studies of viruses and bacteria, which achieve their cytoplasmic entry by rupturing intracellular vesicles, have utilized the redistribution of galectin proteins as a tool to measure vesicle rupture by these organisms. Using this approach, we demonstrate that α-synuclein aggregates can induce the rupture of lysosomes following their endocytosis in neuronal cell lines. This rupture can be induced by the addition of α-synuclein aggregates directly into cells as well as by cell-to-cell transfer of α-synuclein. We also observe that lysosomal rupture by α-synuclein induces a cathepsin B dependent increase in reactive oxygen species (ROS) in target cells. Finally, we observe that α-synuclein aggregates can induce inflammasome activation in THP-1 cells. Lysosomal rupture is known to induce mitochondrial dysfunction and inflammation, both of which are well established aspects of Parkinson's disease, thus connecting these aspects of Parkinson's disease to the propagation of α-synuclein pathology in cells.

## Introduction

Numerous studies have found that alpha-synuclein (α-synuclein) oligomers or small aggregates are toxic to cells in culture and can be spread from affected neurons to neighboring cells [1], [2], [3], [4]. A better understanding of the mechanism by which α-synuclein induces a cellular pathology which is spread to neighboring cells might lead to a better understanding of how Parkinson's disease (PD) pathology spreads *in vivo* [5].

Biochemical studies of α-synuclein have demonstrated that α-synuclein adopts an amphipathic alpha-helix which associates with lipid bilayers [6]. The association of α-synuclein with lipid bilayers induces membrane curvature, tubulation, and disruption [7], [8]. These observations are remarkably similar to our previous studies of adenovirus protein VI. As a non-enveloped virus, adenovirus enters the cytoplasm of target cells via protein VI mediated rupture of endocytic vesicles following internalization [9]. Notably, protein VI also adopts an amphipathic alpha-helix which is capable of disrupting and tubulating artificial liposomes [10]. In addition to facilitating subsequent steps in infection, this rupture of endocytic vesicles by adenovirus is sensed by the target cell as a “danger signal”, resulting in a cathepsin B dependent increase in cellular reactive oxygen species (ROS) [11] and inflammasome activation [12], [13]. In this regard, studies exploring the pathological potential of α-synuclein oligomers or small aggregates have demonstrated that α-synuclein oligomers can be internalized via endocytic pathways [14], [15]. However, it remains unclear how, following endocytosis, α-synuclein escapes the endosome and what relationship this escape may have to the pathology induced by α-synuclein *in vitro* or PD pathology *in vivo* [5].

Given the similarities between α-synuclein and protein VI, we hypothesized that α-synuclein could generate a similar rupture of intracellular vesicles and induce ROS production in cells. Indeed, we observe that the addition of α-synuclein aggregates to neuronal cell lines induces the rupture of endocytic vesicles, specifically lysosomes, in these cells. This rupture induces a cathepsin B dependent increase in ROS in these cells. Large aggregates which were too large to enter cells via endocytosis, did not induce the same degree of vesicle rupture or ROS induction. Finally, using the monocytic leukemic cell line THP-1 as a model of microglial cells, we show that α-synuclein can induce inflammasome activation in these cells. Collectively, these experiments define a pathway by which pathology may be induced in cells during the spread of α-synuclein associated pathology observed in PD.

## Materials and Methods

### Cell lines and reagents

The human neuroblastoma cell line, SH-SY5Y, and the human monocyte cell line THP-1 were obtained from the American Type Culture Collection (ATCC). The rat dopaminergic neuronal cell line was a kind gift from Dr. Anumantha Kanthasamy [16]. SH-SY5Y cells were maintained and differentiated with 50 mM all _L_–*trans* Retinoic acid every 48–72 hours in Dulbecco's modified Eagle's medium (DMEM) supplemented with 10% fetal bovine serum (FBS)(Hyclone) and 100 IU/mL penicillin, 100 µg/mL streptomycin and 10 µg/mL ciprofloxacin. THP-1 cells were maintained in Roswell Park Memorial Institute (RPMI) 1640 media, supplemented with 10% FBS, 100 IU/ml penicillin, 1 mg/ml streptomycin, 0.25 µg/ml amphotericin B, non-essential amino acids, 1 mM sodium pyruvate, 10 mM HEPES buffer and 2 mM glutamine. The rat dopaminergic cell line N27 was cultured in RPMI media with the same additives used in the DMEM. Cells were maintained in a 37°C incubator with 5% CO_2_.

### α-synuclein

Full length α-synuclein and E46K mutant α-synuclein were purchased (rPeptide) and the lyophilized protein was rehydrated to a concentration of 1 mg/ml. α-synuclein was incubated for 3 days at 37°C in PBS with 100 mM NaCl under constant agitation followed by aliquoting and storage at −80°C. Aggregates were fluorescently labeled with Dylight 488 N-hydroxysuccinimide (NHS)-ester fluorophores (Thermo Scientific), according to the manufacturer's protocol prior to use.

### Western Blot analysis

Purified proteins were separated via SDS-PAGE, proteins were transferred to nitrocellulose membranes and detected by incubation with the primary antibody. The H3C monoclonal antibody developed by Julia George was obtained from the Developmental Studies Hybridoma Bank developed under the auspices of the NICHD and maintained by The University of Iowa, Department of Biology, Iowa City, IA 52242. Secondary antibody conjugated to HRP (Thermo Scientific) was used where necessary, and antibody complexes were detected using SuperSignal West Femto chemiluminescent substrate (Thermo Scientific). Chemiluminescence was measured using a BioRad ChemiDoc XRS imaging system (BioRad).

### Coomassie

Samples were run on a native 10% polyacrylamide gel. Gel was fixed using colloidal coomassie fixative (45% methanol and 10% acetic acid) for 1 hour. Gel was then placed in Coomassie Brilliant Blue G-250 (MP Pharmaceuticals) overnight at room temperature. The following morning the gel was imaged using a BioRad ChemiDoc XRS imaging system (BioRad).

### Glutaraldehyde Cross-linking

Equivalent amounts of aggregated α-synuclein and monomeric α-synuclein were incubated with 0, 1, and 2 µM glutaraldehyde for 15 minutes at room temperature. 1 M glycine was added after 15 minutes to saturate the glutaraldehyde. 2× SDS sample buffer was added and the mixture was boiled for 5 min at 100°C. The samples were then subjected to SDS-PAGE using 4%–15% Tris–HCl gradient gels (Ready Gels, BioRad) and subsequent Western Blot analysis.

### K114

The K114 fluorescent amyloid fibril binding assay was performed in 100 mM glycine buffer, pH 8.5 with 100 µM K114 using an Olis-DM 45 spectrofluorometer. Fluorescence was measured with an excitation wavelength of 380 nm, and emission wavelength of 550 nm with a cutoff established at 530 nm for all assay endpoints.

### Immunofluorescence microscopy

Cells were allowed to adhere to Fibronectin (Sigma-Aldrich) treated glass coverslips and fixed with 3.7% formaldehyde (Polysciences) in 0.1 M piperazine-N, N'bis(2-ethanesulfonic acid) PIPES buffer at pH 6.8 for 15 min. Cells were then permeabilized for 20 minutes in PBS with 10% Normal Donkey Serum (NDS) and 0.5% saponin (Sigma-Aldrich). Staining with specific monoclonal or polyclonal antibodies was done in 10% NDS with 0.5% saponin for 20 minutes. Mouse-anti α-synuclein (BD Biosciences), EEA-1 (BD Biosciences), and LAMP-2 (BD Biosciences) were immunostained at 1:400 dilutions. Primary antibodies were secondarily labeled with fluorophore-conjugated donkey anti-mouse or donkey anti-rabbit antibodies (Jackson ImmunoResearch). Images were collected with a DeltaVision microscope (Applied Precision) equipped with a digital camera (CoolSNAP HQ; photometrics), using a 1.4-numerical aperture (NA) 100X objective lens, and were deconvolved with SoftWoRx deconvolution software (Applied Precision). Structured Illumination images were collected on an OMX microscope (Applied Precision) and deconvolved and reconstructed using SoftWoRx software (Applied Precision). Tiff images and 3-dimensional reconstructions were generated using Imaris software (Bitplane).

### Image Analysis

Deconvolved images were analyzed for colocalization of Galectin-3 (Gal-3) with LAMP2 and Early Endosome Antigen-1 (EEA-1) signal by use of the Surpass Mode of the Imaris software package (Bitplane).

### Live Cell Imaging

Images were acquired on a Deltavision deconvolution microscope equipped with a Weather Station™ chamber utilized to maintain the cells at 37° C at 5% CO_2_. Images were acquired using an Cascade 2 EMCCD (Photometrics) and deconvolved as described above.

### ROS Assay

All N27 and SH-SY5Y cells were plated at 100,000–200,000 cells per well in a black 96-well plate (Costar) in RPMI or DMEM with 10% fetal bovine serum (FBS). Following 1 hour of serum starvation the cells were incubated with the ROS-sensitive fluorophore 2, 7-dichlorofluorescein diacetate H_2_DCFDA (DCF; Invitrogen) at 10 µM for 1 hour by following the manufacturer's protocol followed by two washes with phosphate-buffered saline (PBS). If pretreated with cathepsin B inhibitor, cells were incubated with 100 µM CA-074Me (EMD Biosciences) or dimethylformamide (DMF) as vehicle control for 1 hour following DCF incubation. α-synuclein aggregates were added at a concentration of 3 µg/ml. Fluorescence intensity was measured over the course of 72 hours at an excitation wavelength of 485 nm and an emission wavelength of 520 nm on a fluorescent plate reader (Biotek). Results are presented as background subtracted values (background was defined as cells that were not loaded with DCF).

### RoGFP assessment of mitochondrial ROS

RoGFP2 fused to a mitochondrial targeting sequence (mRoGFP) was obtained from S James Remington (University of Oregon). The open reading frame was inserted into lentiviral vectors containing a puromycin resistance cassette and vector stock was used to transduce parental SH-SY5Y cells. Cells stably expressing mRoGFP were selected in DMEM containing 5 µg/ml puromcyin. Cells were imaged in DMEM lacking phenol red (Invitrogen) at 90 second intervals. The basal oxidation state of individual cells was assessed for 6 minutes. 4 mM Dithiothreitol was added after 6 minutes to define the minimum oxidation state of the imaged cells. After 6 minutes (4 time points) in DTT, the cells were washed quickly with PBS and 300 µM Aldrithiol was added to define the maximal oxidation state of the imaged cells. The relative oxidation state of each cell was calculated by fixing the minimum oxidation state observed in the presence of DTT to 0 and the maximum oxidation state observed in the presence of Ald to 1, as previously described {Guzman, 2010 #440}. The average of 15 or more cells from each treatment group were analyzed.

### Quantification of IL-1β secretion by ELISA

THP-1 cells were plated at 200,000 cells per well in a 96-well plate with 5 ng/ml PMA for 48 hours to induce macrophage differentiation. Differentiated macrophages were washed, and then left untreated or pretreated with α-synuclein aggregates for 24 or 48 hours before being serum starved for 2 hours. A subset of cells were then treated with ultrapure LPS (10 ng/ml) for 2 hours, washed , and then left untreated, or treated for 1 hour with 5 mM ATP, as a positive control. Supernatants from each sample were collected, and an ELISA was performed using the Ready-SET-Go! IL-1β kit from eBioscience (catalog no. 88-7010-88).

### Caspase-1 assay

Differentiated THP-1 cells were incubated in the presence or absence of α-synuclein (2.4 µg/ml) for 48 hours before being serum starved for 2 hours, and then left untreated or treated with LPS for 2 hours. Cells were then washed in PBS. Caspase-1 activity in THP-1 cells was assessed with a caspase-1 FLICA kit (Immunochemistry Technologies) according to the manufacturer's instructions. The maximum fluorescent intensity of 50 cells in each treatment group was assessed microscopically on a Deltavision wide field fluorescent microscope (Applied Precision).

### Electron Microscopy

α-synuclein was added to carbon coated, 200mesh grids for 90 seconds. Grids were stained with 2% uranyl acetate for 45 seconds. Grids were dried overnight and imaged on a Hitachi H-600 transmission electron microscope.

## Results

### Characterization of α-synuclein preparations

**Figure 1:**
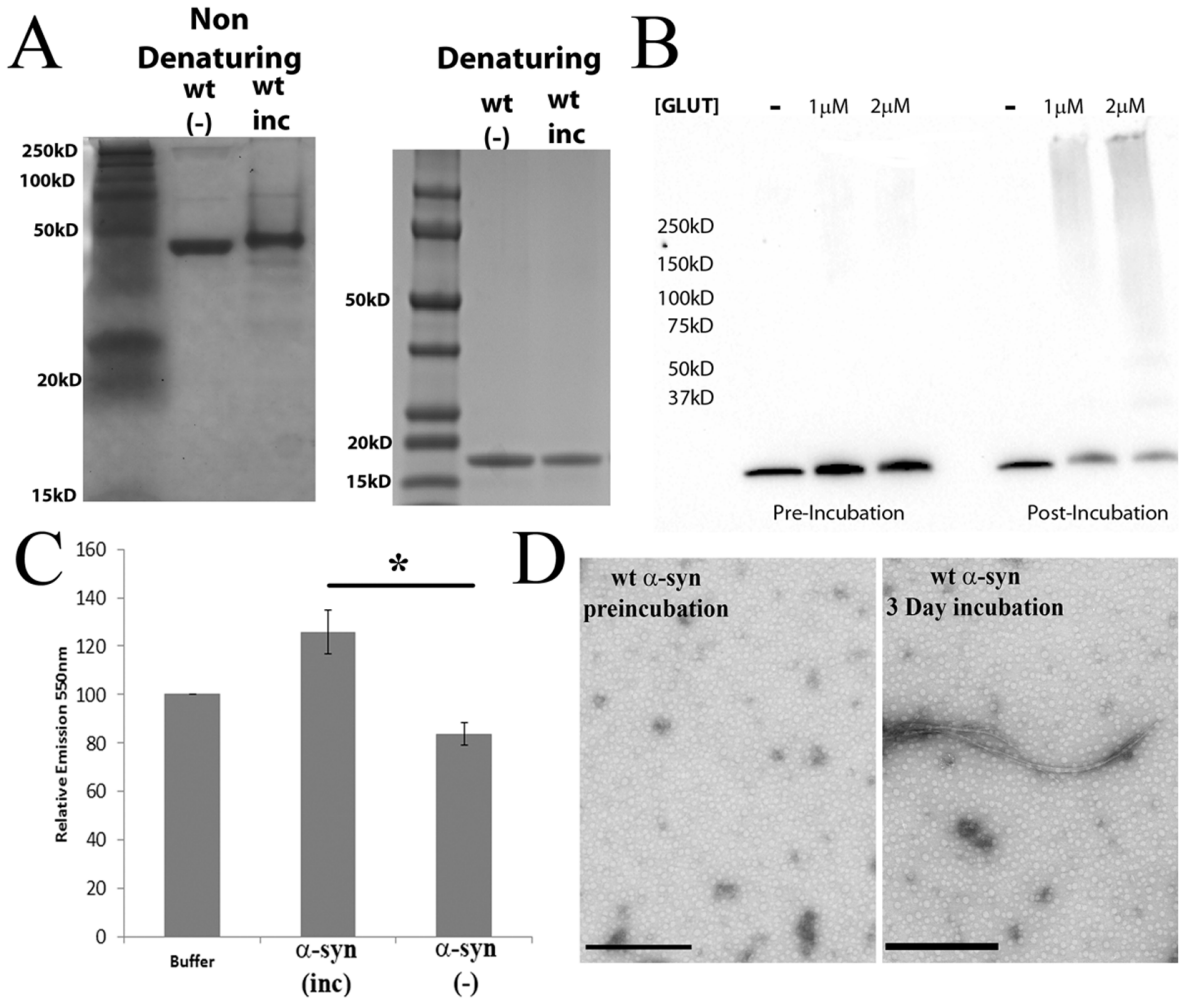
α-synuclein aggregate characterization. A. Wild-type α-synuclein aggregates were generated using *in vitro* purified protein. Recombinant lyophilized α-synuclein was resuspended and followed by constant agitation for three days at 37°C. The aggregates generated in this fashion were run on a non-denaturing gel (left) or denaturing gel (right), fixed and stained with Coomassie brilliant blue. B. Following incubation for 3 days as described, α-synuclein preparations were fixed with glutaraldehyde at the indicated concentration for 15 minutes at room temperature. C. K114 analysis of α-synuclein which was freshly resuspended or incubated as described. Emission was measured at 550 nm. Error bars represent the standard error of the mean of 3 readings. Results are representative of at least three independent experiments (* indicates a P value <.01). D. Transmission electron micrograph of α-synuclein preparations from freshly resuspended protein or after 3 days of incubation as described. Scale bars = 500 nm.

α-synuclein aggregates were generated by resuspending recombinant lyophilized protein followed by agitation for three days at 37°C. Prior to agitation, purified recombinant α-synuclein ran at a molecular weight of slightly less than 50 kD on a non-denaturing gel. Our three day incubation altered the electrophoretic mobility of the protein in non-denaturing gel in two ways. First, the primary band in the solution exhibited less electrophoretic mobility than the presumably monomeric form of the protein (Figure 1A). We also observed the appearance of a number of bands which migrated below the primary band. Both of these changes may represent altered structural changes in α-synuclein induced during incubation or may also represent a multimeric form of α-syn which is induced during incubation. Under denaturing conditions, all of these bands resolved to the expected ∼15 kD molecular weight of α-synuclein (Figure 1A). We also performed glutaraldehyde crosslinking to further define the high molecular weight species of α-synuclein induced by our incubation conditions. Incubation induced the formation of high molecular weight species of α-synuclein observable following glutaraldehyde crosslinking (Figure 1B). These higher molecular weight species were not observed following glutaraldehyde crosslinking of freshly resuspended α-synuclein (Figure 1B). We also characterized our incubated protein with the dye K114, which can be used to measure amyloid fibrils in solution. We observe that incubation induced the formation of fibrillar structures, as measured by an increase in K114 fluorescence relative to freshly resuspended α-synuclein (Figure 1C) (p<0.01). Similarly, transmission electron microscopy of wt α-synuclein (with or without a 3 day incubation at 37°C) revealed fibrillar structures only following incubation. No such structures were detected in the absence of incubation (Figure 1D). These analyses revealed that this protocol induces a heterogeneous combination of fibrillar and oligomeric species of α-synuclein. We will refer to this heterogeneous population of α-synuclein species as “aggregates” in this manuscript.

### α-synuclein aggregates induce the rupture of endocytic vesicles in neuronal cell lines

**Figure 2:**
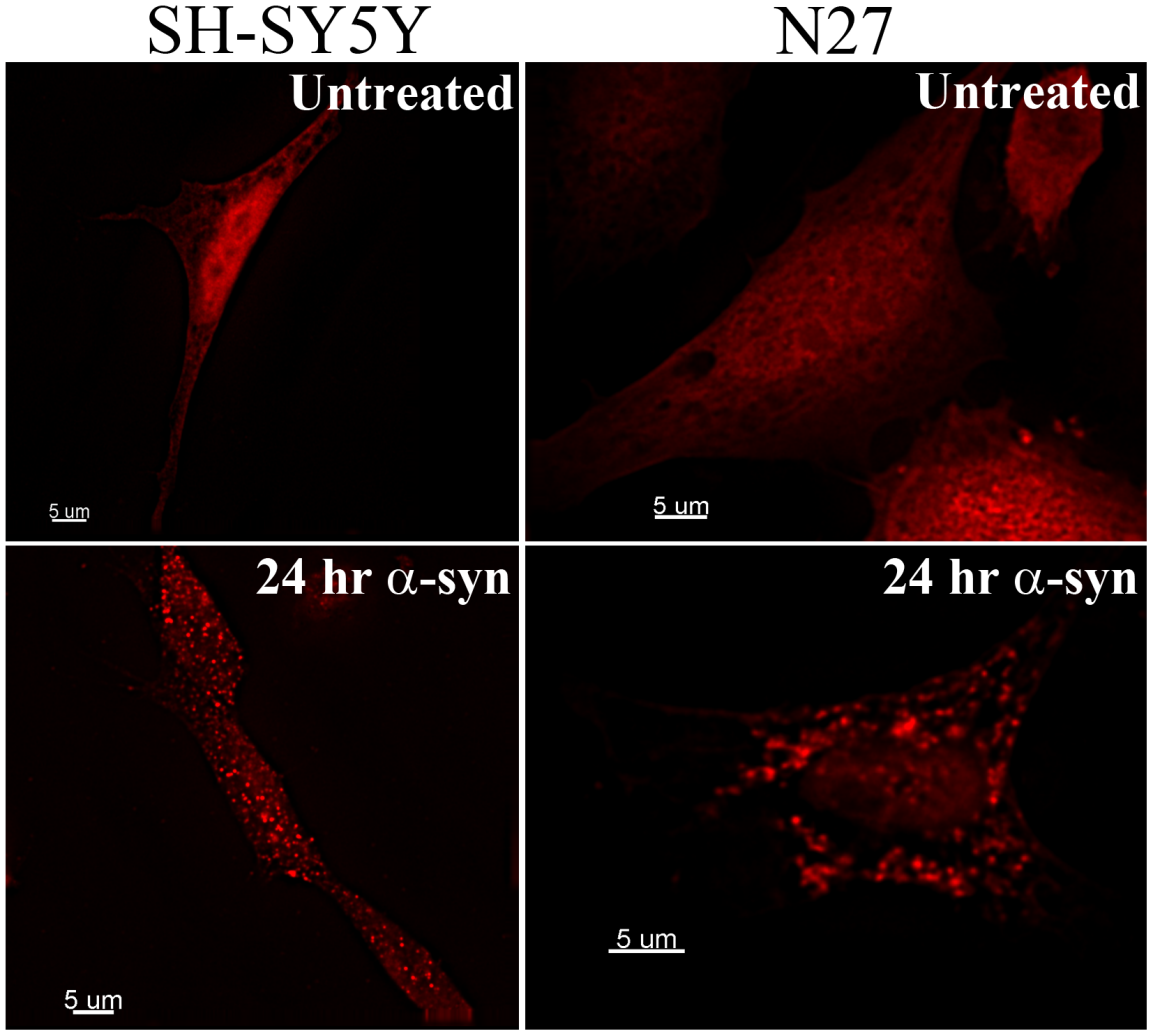
α-synuclein aggregates induce discrete Galectin-3 puncta. SH-SY5Y or N27 cells stably expressing chGal3 were treated with α-synuclein aggregates for 24 hours. In both cell lines a relocalization from the diffuse, untreated state can be clearly visualized after 24 hours. This relocalization is indicative of intracellular vesicular rupture. Images are representative of at least three independent experiments.

Galectin-3 is a sugar binding protein which recognizes beta-galactoside sugars which are normally only present on the exterior leaflet of the plasma membrane and the interior leaflet of intracellular vesicles [17]. Galectin-3 relocalization has therefore been utilized as a tool to identify ruptured vesicles in studies of bacteria and viruses which rely on vesicle rupture to enter the cytoplasm during infection [18], [19], [20]. To test the hypothesis that α-synuclein can induce the rupture of intracellular vesicles following endocytosis, we transduced human SH-SY5Y neuroblastoma cells and the rat dopaminergic neuronal N27 cell line with a retroviral vector expressing mCherry-Galectin3 (chGal3). We treated these cells with the α-synuclein aggregates described in Figure 1. Incubation of N27 or SH-SY5Y cells stably expressing mCherry-Gal3 (N27chGal3, SY5YchGal3) with α-synuclein aggregates induced a pronounced redistribution of chGal3 in both cell lines. While in the absence of treatment chGal3 assumed a diffuse cytoplasmic localization in both cell lines; incubation with α-synuclein aggregates for 24 hours induced the relocalization of chGal3 to intracellular, punctate structures (Figure 2). This relocalization suggests that α-synuclein aggregates are able to disrupt the integrity of vesicular membranes following endocytosis. Treatment of cells with freshly resuspended α-synuclein did not induce relocalization of chGal3 (Figure S1), suggesting that non-monomeric forms of α-synuclein are responsible for vesicular rupture under these conditions.

### α-synuclein aggregates induce the rupture of lysosomes following endocytosis

**Figure 3:**
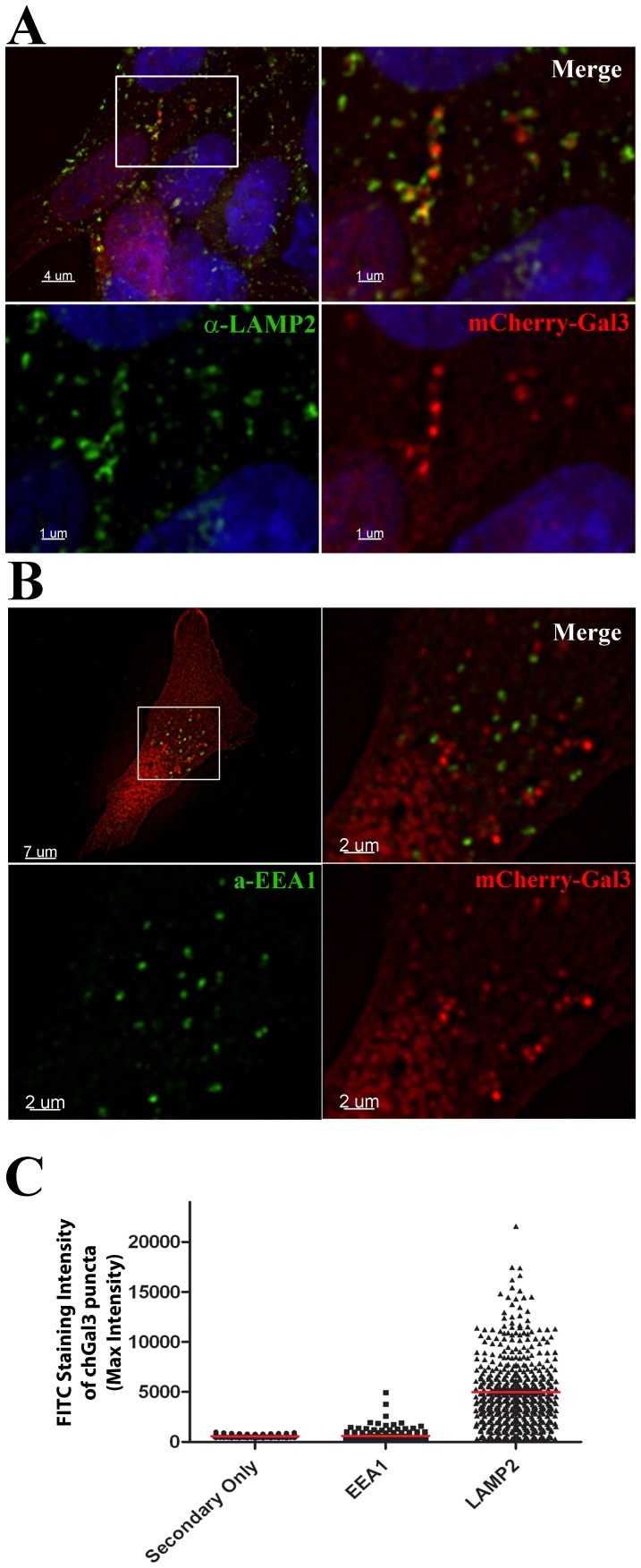
α-synuclein aggregates induce lysosomal rupture. A. SH-SY5Y chGal3 cells (red) treated with α-synuclein aggregates hibit colocalization between chGal3 and LAMP2 (green). The area shown in the white box in the upper left panel is enlarged in the other three panels. B. SH-SY5Y chGal3 cells (red) treated with α-synuclein, which does not induce colocalization between chGal3 and EEA1 (green). The area shown in the white box in the upper left panel is enlarged in the other three panels. C. Quantitative analysis of the colocalization of fluorescent intensity of EEA1 or LAMP2 staining in individual chGal3 puncta identified using Imaris image analysis software. 7.43% of chGal3-positive vesicles exhibited EEA1 staining above background levels, whereas 91.98% of these vesicles exhibited LAMP2 staining greater than secondary antibody alone. Forty images were collected for each group. More than 190 individual puncta were analyzed for each group.

Our previous studies of adenovirus found that protein VI mediated vesicle rupture occurs more frequently in vesicles positive for the endosomal marker Early Endosome Antigen 1 (EEA1), than in those positive for the lysosomal marker, Lysosomal Assosiated Membrane Protein-1 (LAMP-1) [18]. We similarly assessed the nature of the ruptured vesicles induced following α-synuclein incubation. Unlike the case of vesicles ruptured by protein VI, we observed the vast majority of chGal3 positive vesicles induced following incubation with α-synuclein aggregates were positive for the lysosomal marker, LAMP2 (Figure 3A), while very few chGal3 positive vesicles showed EEA1 staining above background (Figure 3B). This observation was confirmed by quantitative analysis of the amount of LAMP2 or EEA1 signal present in individual chGal3 puncta (7.43% of chGal3-positive vesicles exhibited EEA1 staining above background levels, whereas 91.98% of these vesicles exhibited LAMP2 staining greater than secondary antibody alone (Figure 3C). This suggests that, following endocytosis, α-synuclein induces the rupture of vesicles which are predominantly lysosomes, in contrast to the case of protein VI, which more frequently ruptures vesicles containing positive for EEA1 [18].

### α-synuclein localizes to areas of vesicular rupture in cells

**Figure 4:**
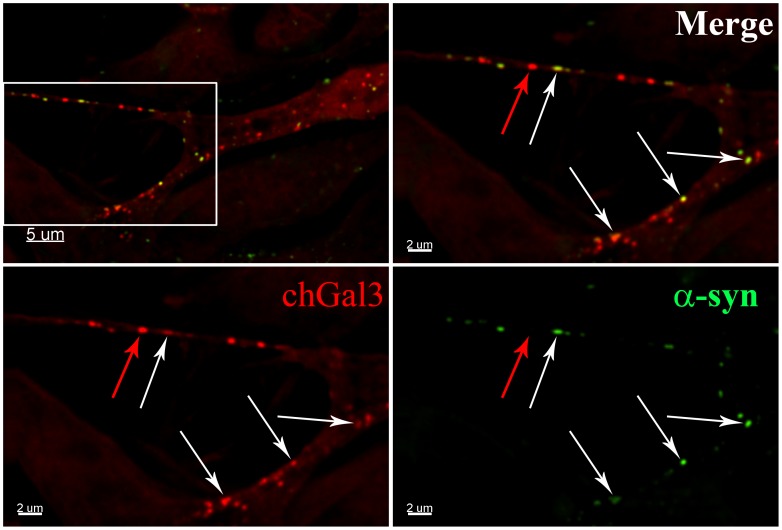
α-synuclein colocalizes with chGal3 in neuronal cell lines. SH-SY5Y chGal3 cell exposed to Dylight 488 conjugated α-synuclein aggregates for 24 hours. The white box in the upper left panel is enlarged in the other three panels to demonstrate colocalization of α-syn and chGal3. White arrows show vesicles demonstrating colocalization between chGal3 and α-syn. The red arrow highlights a ruptured vesicle that does not appear to contain α-synuclein.

We next determined if α-synuclein localized to ruptured vesicles by directly labeling α-synuclein aggregates with the Dylight 488 N-hydroxysuccinimide (NHS)-ester [21]. When fluorescently labeled α-synuclein aggregates were added to SY5Y chGal3 cells, ruptured vesicles containing α-synuclein were clearly evident in these cells 24 hours later (White Arrows, Figure 4A). However, we also observed chGal3 puncta which did not contain detectable α-synuclein signal (Red Arrow, Figure 4A). This association of α-synuclein with ruptured vesicles was confirmed in live cell imaging experiments, in which α-synuclein and chGal3 remain associated during brief intracellular trafficking events (Movie S1).

**Figure 5:**
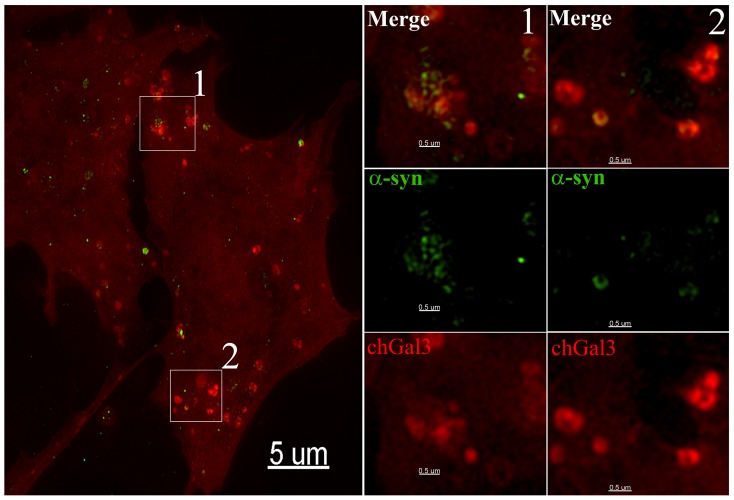
Structured Illumination (SIM) imaging of α-synuclein mediated vesicle rupture. N27chGal3 cells were exposed to Dylight 488 conjugated α-synuclein aggregates for 48 hours. Cells were fixed and imaged using a structured illumination fluorescent microscope, allowing the images to be reconstructed with a resolution below the diffraction limit of light microscopy. The boxed area in the left panel is enlarged in the two panels to reveal the intraluminal localization of α-synuclein.

To better assess the localization of α-synuclein within ruptured vesicles, we performed structured illumination microscopy (SIM). SIM utilizes pattern based imaging of a specimen to generate reconstructions of the specimen with resolutions below the diffraction limit of light microscopy (∼200 nm) [22]. When reconstructions of N27 cells incubated with α-synuclein were analyzed, α-synuclein was frequently observed to associate with the periphery of chGal3 positive vesicles, frequently forming arced or circular localizations (Figure 5). In some cases, α-synuclein formed an individual arc at the periphery of discrete ruptured vesicles (panel 2, Figure 5). In other cases, α-synuclein clustered in a series of circular localizations suggesting the presence of α-synuclein in a multivesicular compartment (panel 1, Figure 5). Additional examples of the intravesicular localization of α-synuclein are shown in Figure S2. The arced localization of α-synuclein at the vesicular periphery was also evident in three dimensional modeling of these reconstructions (Movie S2)._This is consistent with the idea that luminal α-synuclein induces membrane curvature that causes the rupture of these vesicles. Collectively these results demonstrate that following entry into target cells, α-synuclein aggregates are present in endocytic vesicles which have been ruptured. As these vesicles are not present in the absence of α-synuclein aggregates, we conclude that α-synuclein mediates the rupture of these vesicles.

### Cell to cell transfer of α-synuclein induces vesicular rupture

**Figure 6:**
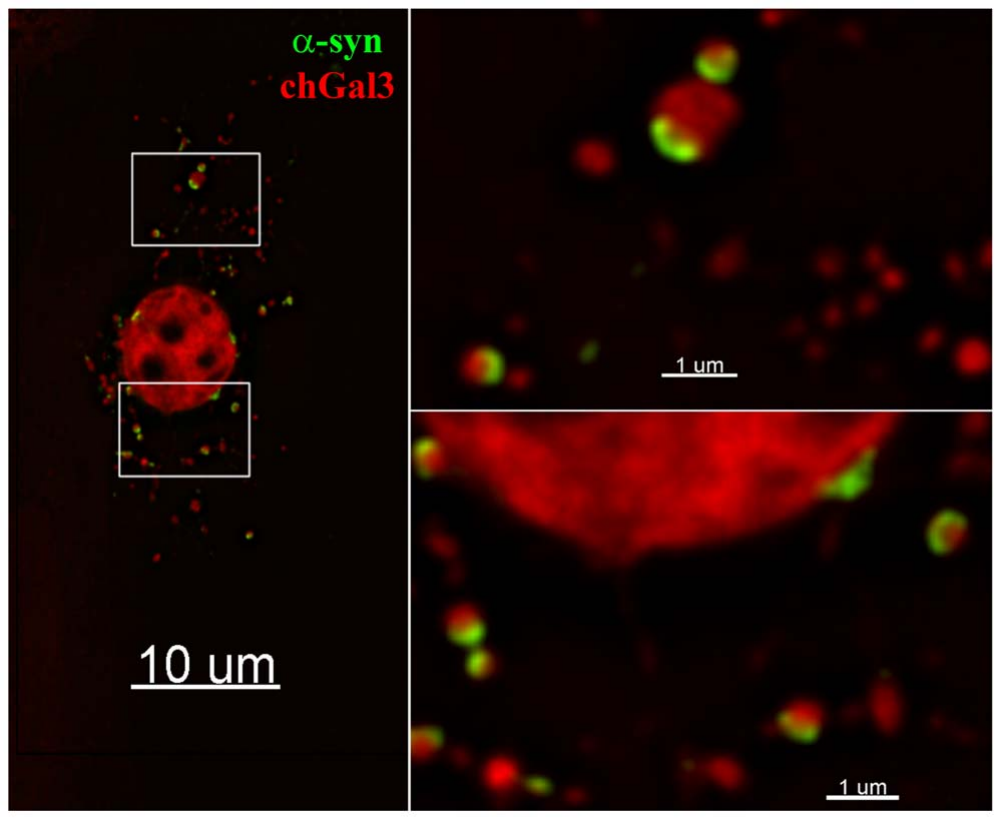
Vesicle rupture following the cell to cell transfer of α-synuclein. N27 cells stably expressing α-synuclein and treated with MPP^+^ for 24 hours. N27chGal3 cells were then added to the culture and co-cultured for 48 hours. Cells were then fixed and stained for α-synuclein (green). The boxed areas in the left panel are enlarged to allow visualization of α-synuclein and chGal3 colocalization.

To determine whether α-synuclein expressed from one cell could induce the rupture of vesicles in neighboring cells, we treated N27 cells stably expressing α-synuclein (N27 α-synuclein) with the mitochondrial toxin MPP^+^ (1-methyl-4-phenylpyridine), which is used to induce a Parkinson's like pathology in tissue culture [23]. Following MPP^+^ treatment, we added N27chGal3 cells to the culture and incubated these cells together for a period of 48 hours. In this mixed culture, colocalization of α-synuclein and chGal3 could indeed be observed. In the example shown in Figure 6, α-synuclein can be observed localizing to crescent shaped structures at the periphery of chGal3 positive vesicles. We therefore conclude that α-synuclein expressed and released from eukaryotic cells can subsequently be internalized and induce vesicle rupture in a manner similar to what we observed with α-synuclein aggregates generated with recombinant protein.

### α-synuclein aggregates induce a cathepsin dependent increase in reactive oxygen species in cells

**Figure 7:**
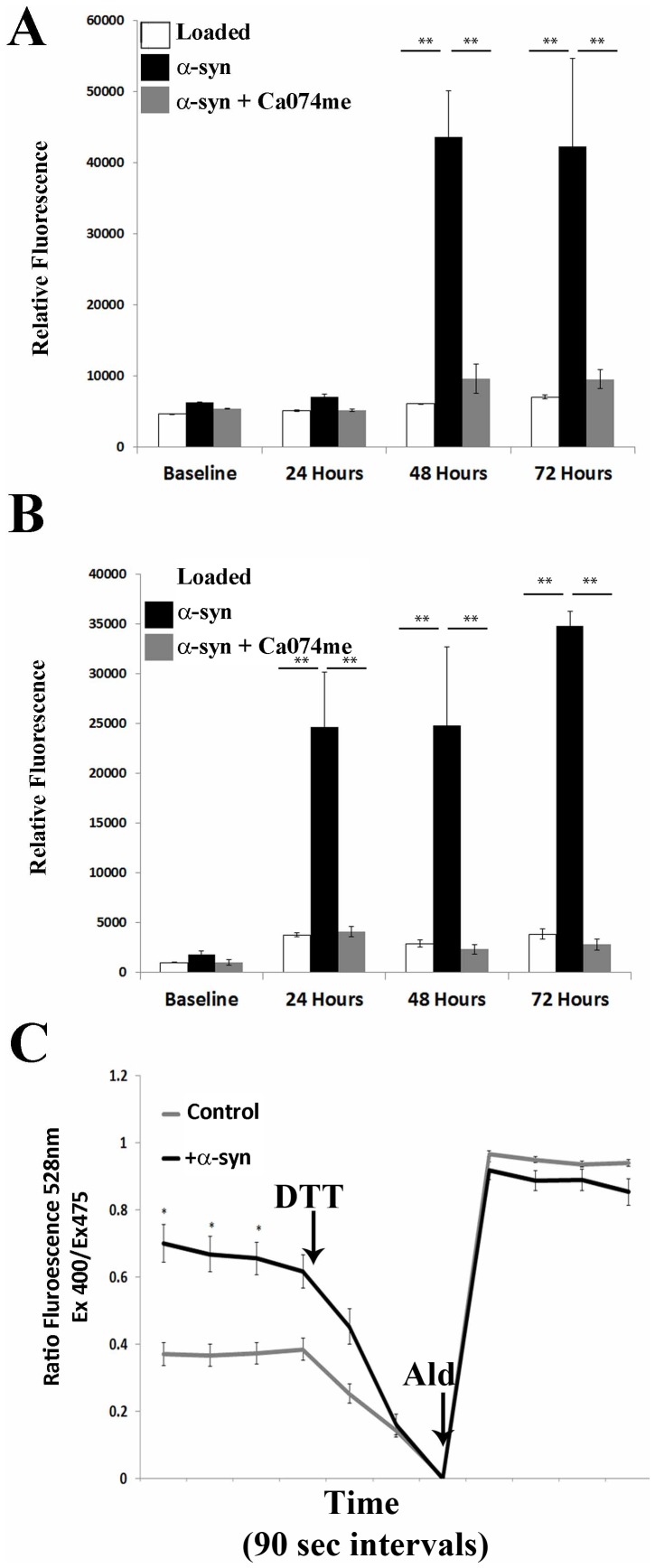
α-synuclein aggregates induce a cathepsin B dependent increase in ROS in cells. SH-SY5Y (A) or N27 (B) cells loaded with the fluorescent substrate H_2_DCFDA reagent and incubated in the presence or absence of α-synuclein aggregates with or without treatment with the cathepsin B inhibitor Ca-074Me. Error bars represent the SEM of triplicate samples. C. SH-SY5Y cells stably expressing mitochondrially targeted roGFP1 were incubated for 72 hours with α-synuclein aggregates and the oxidation state of the mitochondria of individual cells was assessed at described in the text. Error bars represent the SEM of 15 or more cells analyzed for each treatment group. * p<0.001.

The rupture of endosomes and lysosomes is associated with an increase in cellular ROS in cells [11], [24], [25], [26]. We therefore asked if vesicle rupture by α-synuclein aggregates could similarly induce an increase in ROS in cells. We utilized the redox sensitive fluorophore H_2_DCFDA to monitor the ROS generated in neuronal cells following treatment with α-synuclein aggregates. Treatment with α-synuclein aggregates induced a significant (P<0.05 1-way ANOVA with Bonferroni post-hoc) increase in the fluorescence observed in both the N27 and SH-SY5Y cells (Figure 7A, 7B). To determine if this increase in ROS was related to the release of activated cathepsins from ruptured lysosomes, we incubated cells with α-synuclein aggregates in the presence of the cathepsin B inhibitor, Ca074-me. Ca074-me significantly abrogated the increase (P<0.01) in ROS observed following treatment with α-synuclein aggregates (Figure 7A, 7B). These experiments collectively demonstrate that α-synuclein can induce a cathepsin B dependent increase in ROS in target cells.

In order to corroborate this data, we utilized SH-SY5Y cells stably expressing a redox sensitive form of GFP [27], [28] targeted to mitochondria to determine if the ROS induced by α-synuclein specifically altered the redox state of the mitochondrial membrane in affected cells. This protein allows for the measurement of the mitochondrial oxidation state in living cells relative to the minimum oxidation, which is measured by the addition of a strong reductant, dithiothrietol (DTT), and to the maximum oxidation, which is measured by the addition of a strong oxidant, aldrithiol [29]. The initial oxidation state for each cell can then be calculated relative to the minimal and maximal oxidation states defined by these two conditions. As shown in Figure 7C, cells treated with α-synuclein aggregates exhibited increased mitochondrial oxidation relative to control treated cells.

### Larger aggregates generated by the E46K α-synuclein variant do not induce vesicular rupture and induce attenuated ROS production in target cells

We next examined the ability of the genetically-linked PD α-synuclein mutant, E46K, to induce vesicular rupture and ROS in target cells. When purified, recombinant E46K α-synuclein was subjected to the same incubation protocol as wild-type (wt) the E46K mutant exhibited reduced electrophoretic mobility compared to wt aggregates (Figure S3A). As with the wt, all of these bands resolved to the expected ∼15kD band under denaturing conditions (data not shown). Glutaraldehyde crosslinking revealed that higher molecular weight species of E46K α-synuclein were also present following incubation (Figure S3B). K114 analysis revealed that the E46K mutant had more fibrillar content that wt α-synuclein incubated under the same conditions (p<0.01) (Figure S3C). Consistent with this observation, macroscopically visible aggregates were present in the E46K samples following incubation (data not shown). Fibrillar species were also readily detectable in these preparations using electron microscopy (Figure S3D). Thus, in following our aggregation protocol, E46K α-synuclein formed fibrillar structures to a greater degree than that observed for wt α-synuclein.

**Figure 8:**
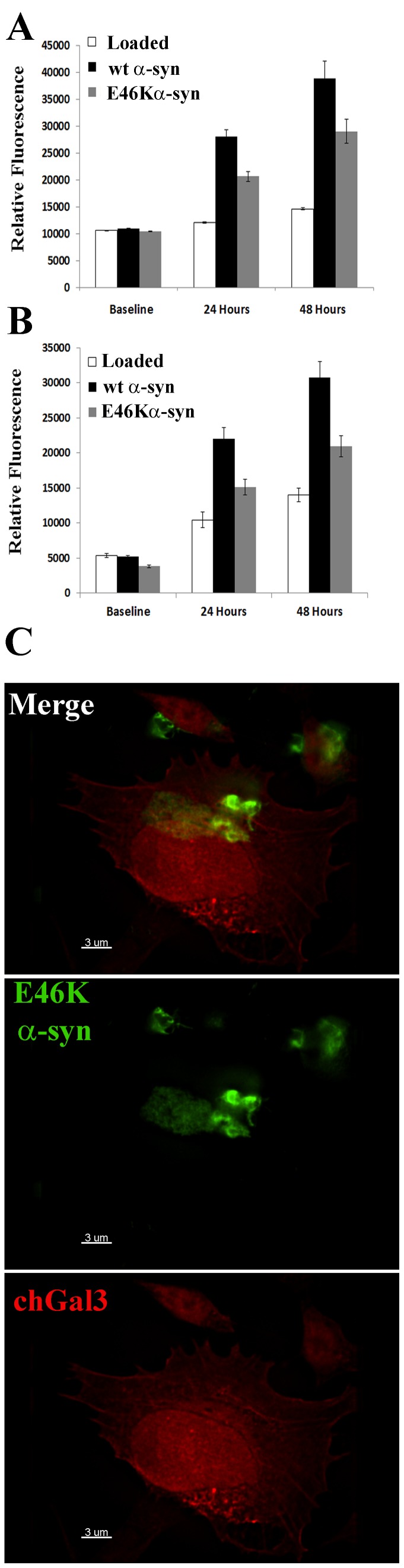
Large aggregates generated by the E46K mutant of α-synuclein do not induce vesicle rupture or ROS as efficiently as wt α-synuclein. (A) SH-SY5Y or (B) N27 cells were loaded with H_2_DCFDA reagent and incubated with wt or E46K α-synuclein. Error bars represent the SEM of triplicate samples. Results are representative of at least three independent experiments. C. Purified recombinant E46K α-synuclein aggregates were added to N27 chGal3 cells for 24 hours, stained with an α-synuclein specific antibody (green) and imaged as in previous experiments. Results are representative of at least three independent experiments.

We next asked if E46K α-synuclein preparations were as competent at wt α-synuclein to induce ROS in target cells. E46K α-synuclein aggregates induced significantly less ROS than wt α-synuclein in N27 and SH-SY5Y cells (Figure 8A, 8B), (p = 0.039 in N27, p = 0.024 in SY5Y). However, this degree of ROS induction was still significant relative to control treated cells (p<0.001 in N27 p<0.016 in SY5Y). When cells treated with these E46K α-synuclein aggregates were analyzed by fluorescent microscopy, we observed that a significant amount of the α-synuclein stain localized to large aggregates that appeared too large to enter the cell via endocytosis. These aggregates did not induce chGal3 relocalization (Figure 8C). Collectively, these results demonstrate that large α-synuclein amyloid aggregates do not induce chGal3 relocalization and preparations which contain larger protein aggregates are less effective at inducing ROS in target cells. The most likely explanation for this observation is that large aggregates are unable to enter the endocytic compartment and therefore do not induce the rupture of lysosomes and subsequent cathepsin dependent mitochondrial stress.

### α-synuclein aggregates induce inflammasome activation in THP-1 cells

**Figure 9:**
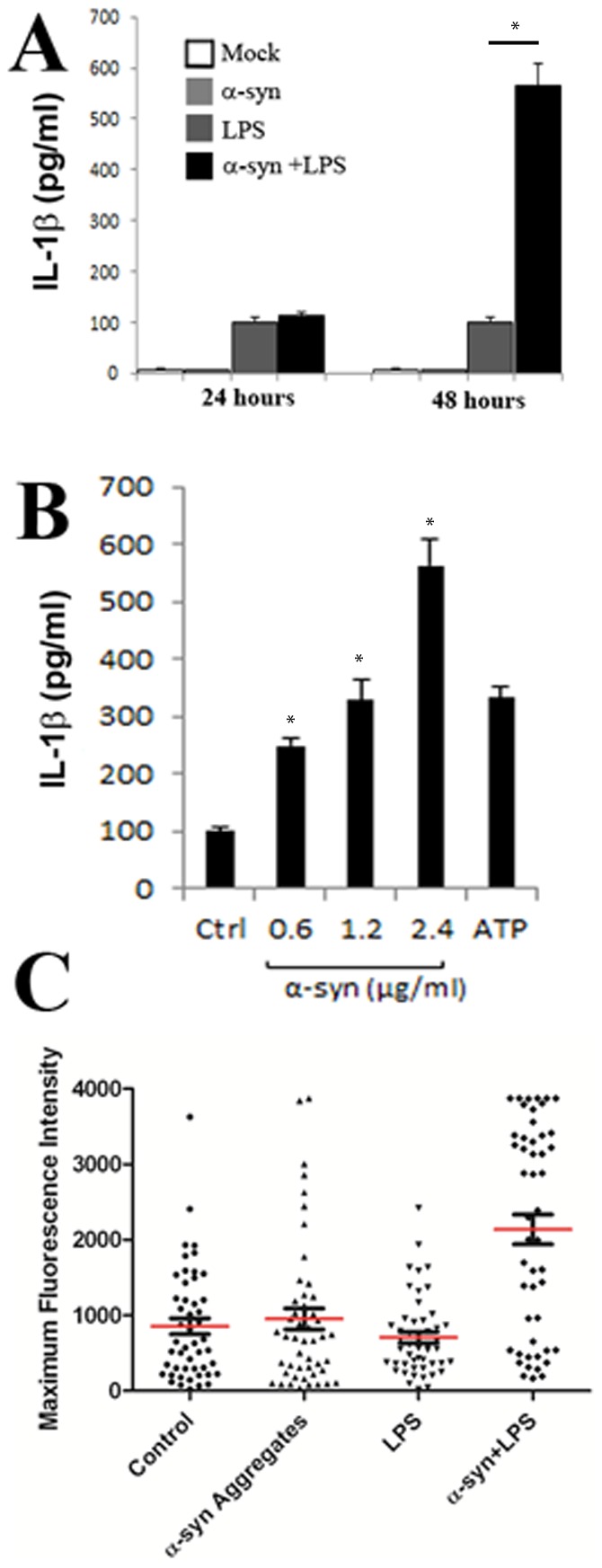
α-synuclein aggregates induce the caspase-1-dependent release of interleukin-1β. A. ELISA of the time dependent release of IL-1β by differentiated THP-1 cells left untreated (Mock) or stimulated with α-synuclein (2.4 µg/ml) and/or LPS. B. ELISA of the dose dependent release of IL-1β into supernatants of LPS-primed THP-1 cells left unstimulated (Ctrl) or stimulated with an increasing amount of α-synuclein, or ATP (5 mM). C. Quantification of caspase-1 activation in THP-1 cells. Caspase-1 activation was visualized by incubation with a fluorescent cell-permeable probe that binds only to activated caspase-1 (FLICA). Data represents the means and standard errors from 3 replicates. *p<0.01

It is known that the rupture of endocytic vesicles by adenovirus [12], [13], silica crystals, [30] or amyloid beta [26] can induce inflammasome activation in macrophages and microglial cells, resulting in the release of pro-inflammatory cytokines IL-1β and IL-18. Inflammasome activation culminates in the activation of caspase-1, which cleaves pro-IL-1β and IL-18 into their active, secreted form [31], [32]. We similarly asked if α-synuclein induced vesicle rupture could induce inflammasome activation in LPS primed THP-1 cells. THP-1 cells are a human leukemic cell line, which are frequently used as a microglial model system following their differentiation with phorbol myristate acetate (PMA) [33]. As shown in Figure 9A, incubation of PMA differentiated THP-1 cells with α-synuclein aggregates or LPS alone did not induce substantial IL-1β expression after 24 or 48 hours. However, the presence of both α-synuclein and LPS induced potent inflammasome activation 48 hours following treatment (p<0.01). Increasing amounts of α-synuclein aggregates induced a proportional increase in the amount of IL-1β released from these cells 48 hours after aggregate addition (Figure 9B). To demonstrate that the release of IL-1β was a result of caspase-1 activation, we cultured PMA differentiated THP-1 cells in the presence or absence of LPS and/or α-synuclein aggregates and measured caspase-1 activation using the fluorometric caspase-1 substrate FLICA. The fluorescent intensity of individual cells was assessed microscopically for individual cells of each treatment group. As observed with IL-1β release, FLICA fluorescence was highest in cells treated with both LPS and α-synuclein as compared to control (Figure 8C), (p<0.01). Collectively, these experiments demonstrate that α-synuclein induced vesicle rupture can induce inflammasome activation in differentiated THP-1 cells.

## Discussion

In this study, we tested the hypothesis that α-synuclein aggregates induce the rupture of vesicles of the endosome/lysosome pathway. This hypothesis was founded on the observation that α-synuclein and adenovirus protein VI can both induce the tubulation and rupture of synthetic membranes [8], [10]. Given that protein VI is the viral protein responsible for inducing vesicle rupture during infection [9], we hypothesized that α-synuclein may possess a similar activity that is relevant to its pathology. Utilizing the relocalization of Galectin-3 as a marker of vesicular rupture [18], [19], [20], [34], we demonstrate that α-synuclein aggregates can induce the rupture of vesicles in the neuronal cell lines N27 and SH-SY5Y. We demonstrate that the ruptured vesicles induced by α-synuclein are positive for LAMP2, and that the appearance of these ruptured vesicles correlates with the induction of a cathepsin B dependent increase in ROS in these cells (Figure 7).

Previous studies have demonstrated that adenovirus trafficking within, and eventual escape from, specific endosomal compartments is largely determined by the receptors used for cell entry [12], [35], [36]. For human adenovirus 5, several groups have demonstrated that protein VI release from the capsid involves mechanical capsid uncoating [37] and release of protein VI at, or near, the cell surface [18], [37], [38]. We have previously shown that this early release of protein VI correlates with the rupture of cell membranes prior to, or upon reaching early endosomes [18]. Early escape from endosomes may be favorable for human adenovirus 5, as it results in the release of fewer activated cathepsins into the cytosol and a reduced inflammatory response compared to other human adenovirus serotypes that enter cells by using different receptors [12]. Conversely, vesicle rupture by α-synuclein is likely not a property that provides selective advantage to the organism. Rather, it seems likely that this property may be an indirect and evolutionarily unintended activity of a protein that has likely evolved to perform a function other than vesicle rupture. Although it is possible that the α-synuclein aggregates used in our study do not precisely recapitulate the ability of toxic α-synuclein species to mediate vesicle rupture in PD, a number of other *in vitro* and *in vivo* studies collectively suggest lysosomal dysfunction may be a central aspect of PD pathology (reviewed in [39]).

The Lansbury group has demonstrated that α-synuclein can induce both small membrane pores on artificial membranes, which allows for the diffusion of ions and very small molecules, as well as a more dramatic permeabilization at higher concentrations, which allows much larger molecules and proteins to pass through the lipid bilayer [40], [41]. Although our studies do not directly compare these two possibilities, the molecular weight of the mCherry-Gal3 fusion protein is much larger than the size limit of pore diffusion reported in these studies, suggesting that membrane rupture, rather than pore formation, is required for the localization of chGal3 to vesicles ruptured by α-synuclein.

Biochemical analysis of the α-synuclein aggregates utilized in this study reveals the presence of both fibrillar and oliomeric species of α-synuclein (Figure 1). Future studies are required to determine which forms of α-synuclein mediate vesicular rupture most efficiently._The aggregation prone α-synuclein variant E46K, which tended to form aggregates too large to enter the endocytic compartment, did not induce ruptured vesicles to the same degree as wt α-synuclein, nor did it induce ROS levels equivalent to wt α-synuclein (Figure 8). This does not demonstrate that E46K α-synuclein has less pathological potential than wt α-synuclein. In fact, the E46K mutation is linked to familial PD [42]. However, in the context of these experiments, we suspect that the tendency to self-associate *in vivo* into pathological species of α-synuclein is mimicked by the protocols employed here to induce the aggregatation of wt α-synuclein. When the E46K mutant was subjected to similar protocols, much larger species of aggregates were induced which allowed us to compare the relative pathological potential of large aggregates and small aggregates in our experimental system. This is consistent with a previous report suggesting that E46K has an increased propensity to assemble into larger, insoluble fibrils with an amyloid architecture [43]. We cannot exclude the possibility that the reduced vesicular rupture and ROS induction by the E46K mutant is due to the E46K mutation. However, we favor the idea that these larger aggregates induced with the E46K variant were less toxic because they were too large to be internalized into target cells, preventing them from inducing the rupture of intracellular vesicles.

The fact that chGal3 positive ruptured vesicles do not ubiquitously contain detectable amounts of α-synuclein suggests that α-synuclein has the capacity to dissociate from the endocytic vesicle following rupture, although we cannot exclude the possibility that these vesicles contained α-synuclein which was not detected in our experiments. Similarly, not all of the α-synuclein signal we observed colocalized with chGal3. This may α-synuclein which has dissociated from a vesicle following rupture or alternatively may be α-synuclein still existing within the vesicular compartment which has not induced vesicle rupture. While we cannot distinguish between these possibilities using this assay, the observation that α-synuclein localizes to the periphery of ruptured vesicles is consistent with the idea that α-synuclein induces membrane curvature capable of inducing the rupture of these vesicles.

It is also worth noting how the pathological pathway identified here might be relevant to the propagation of α-synuclein pathology *in vivo*. Work from other labs, taken with the data presented here, suggests a mechanism by which α-synuclein mediated lysosome rupture may perpetuate the propagation of α-synuclein pathology. Specifically, Alvarez-Erviti and coworkers have demonstrated that lysosomal dysfunction and stress increase the release of α-synuclein containing exosomes [44]. Danzer and colleagues have demonstrated α-synuclein containing exosomes induce more pathology than recombinant aggregates [45]. Taken together, the observations here and in these studies suggest a mechanism by which continued vesicular rupture by α-synuclein may not only exert toxic effects on a given cell but may also perpetuate the propagation of α-synuclein pathology to neighboring cells.

Although the data presented here utilize cell lines to demonstrate lysosomal rupture and ROS induction, it is worth nothing that others have reported clinical observations consistent with the pathway identified. Reduced cathepsin and LAMP immunoreactivity has been observed in nigral neurons in PD patients [46], consistent with the idea of vesicle rupture and cytoplasmic diffusion of lysosomal contents. The role of mitochondrial dysfunction is also well established in PD [47].

Our observation that α-synuclein can induce inflammasome activation in a microglia like cell line may be relevant to the neuroinflammatory aspects of PD [48], [49]. Notably, two hallmarks of inflammasome activation, IL-1β release [50] and caspase-1 activation [51] have been reported to be elevated in the substantia nigra of PD patients.

Here, we define the pathway by which α-synuclein escapes the vesicular compartment and induces toxicity in tissue culture cells. Future studies are needed to determine the degree to which α-synuclein mediated lysosomal rupture affects the propagation of PD pathology in primary neuronal cultures, animal models and individuals affected by PD and other synucleinopathies.

## Supporting Information

Figure S1 **α-synuclein monomers do not induce chGal3 relocalization.** N27 and SH-SY5Y cells stably expressing chGal3 were incubated with freshly resuspended α-synuclein for 24 hours. Treatment of these cells with freshly resuspended α-synuclein did not induce the redistribution observed at an equivalent concentration of α-synuclein aggregates.
(TIF)

Figure S2 **Intravesicular localization of α-synuclein.** N27chGal3 cells were treated with Dylight 488 conjugated α-synuclein aggregates for 48 hours as described in the text. Shown are ruptured vesicles containing α-synuclein revealing the localization of α-synuclein to the vesicle periphery.
(TIF)

Figure S3 **E46K aggregate characterization.** E46K mutant α-synuclein was generated using in-vitro purified protein. Recombinant lyophilized E46K α-synuclein was resuspended and constantly agitated for three days at 37°C. A. The aggregates generated in this fashion were run on a non-denaturing gel, fixed and stained with Coomassie brilliant blue. E46K α-synuclein ran at a higher molecular weight than the wild-type α-synuclein on the non-denaturing gel. B. Following incubation for 3 days as described, E46K α-synuclein preparations were fixed with glutaraldehyde at the indicated concentration for 15 minutes at room temperature. C. The fibrillar content of E46K aggregates was assessed using K114 staining. E46K aggregates had significantly more fibrillar content than wt aggregates following identical treatement (*P<0.01). Results are representative of at least three independent experiments D. TEM image of E46K α-synuclein fibril.
(TIF)

Movie S1 **α-synuclein aggregates associate with ruptured vesicles.** A. Live-cell movie of SH-SY5Y chGal3 cells that have been exposed to Dylight 488 conjugated α-synuclein aggregates for 24 hours. The chGal3 signal is red, and the labeled α-synuclein aggregates are displayed in green. Images were collected at five second intervals for five minutes.
(WMV)

Movie S2 **3-dimensional reconstruction of α-synuclein localization within a ruptured vesicle.** The cell featured in Figure 4B is shown. As the movie plays, the raw data is gradually replaced with 3-dimensional surfaces created from the 3-dimensional Z-stack SIM reconstruction. Towards the end, the opacity of the featured vesicle is reduced to allow the visualization of α-synuclein within the vesicle, where it can be observed forming an arced localization at the periphery of the vesicle.
(WMV)
