# Supplementary figures and images for: Alpha-Synuclein Induces Lysosomal Rupture and Cathepsin Dependent Reactive Oxygen Species Following Endocytosis
Source: PLoS One. 2013 Apr 25;8(4):e62143. doi: 10.1371/journal.pone.0062143 (PMC3636263; doi:10.1371/journal.pone.0062143)

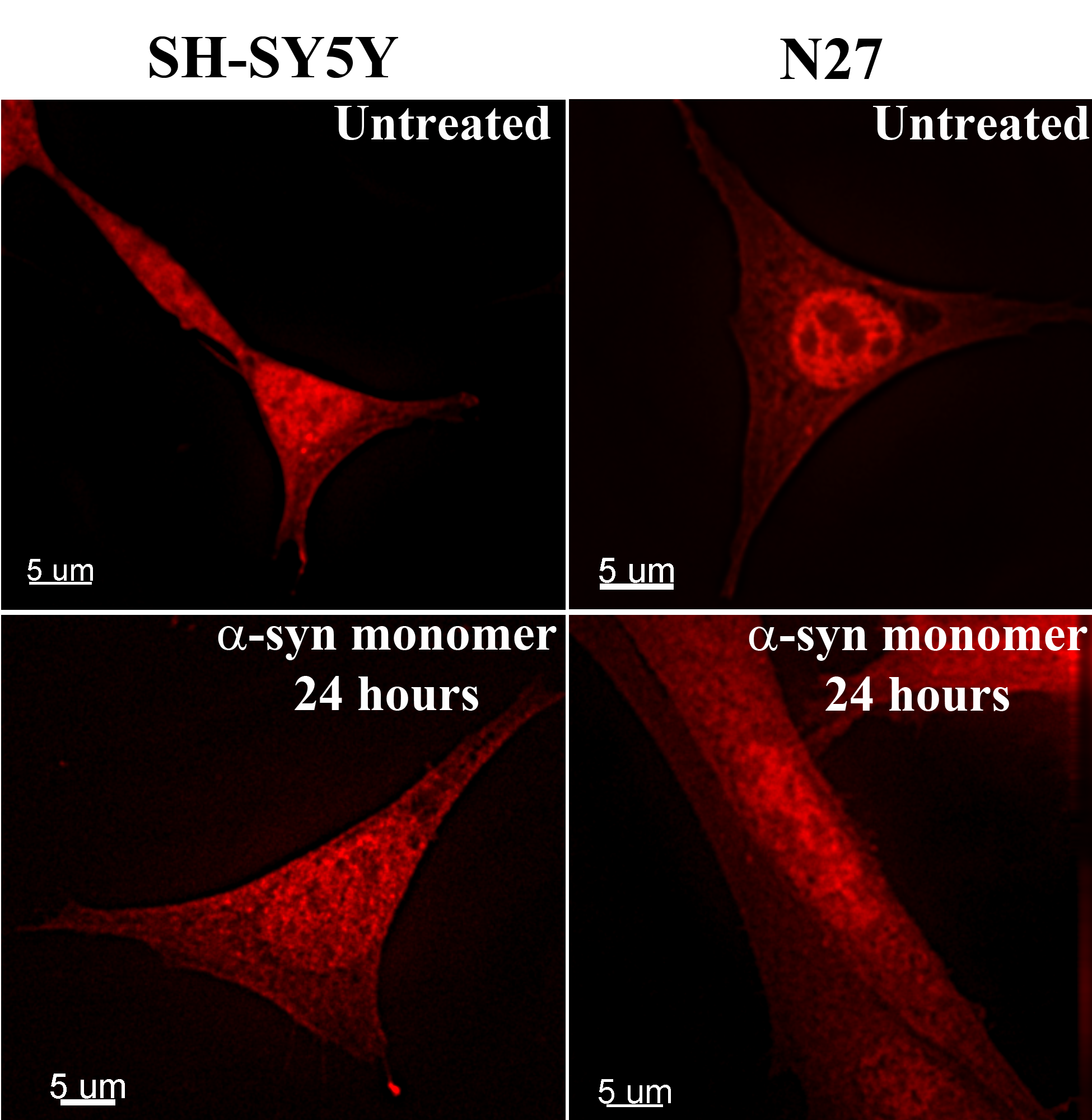

Supplement: Figure S1 — α-synuclein monomers do not induce chGal3 relocalization. N27 and SH-SY5Y cells stably expressing chGal3 were incubated with freshly resuspended α-synuclein for 24 hours. Treatment of these cells with freshly resuspended α-synuclein did not induce the redistribution observed at an equivalent concentration of α-synuclein aggregates. (TIF) [file pone.0062143.s001.tif]

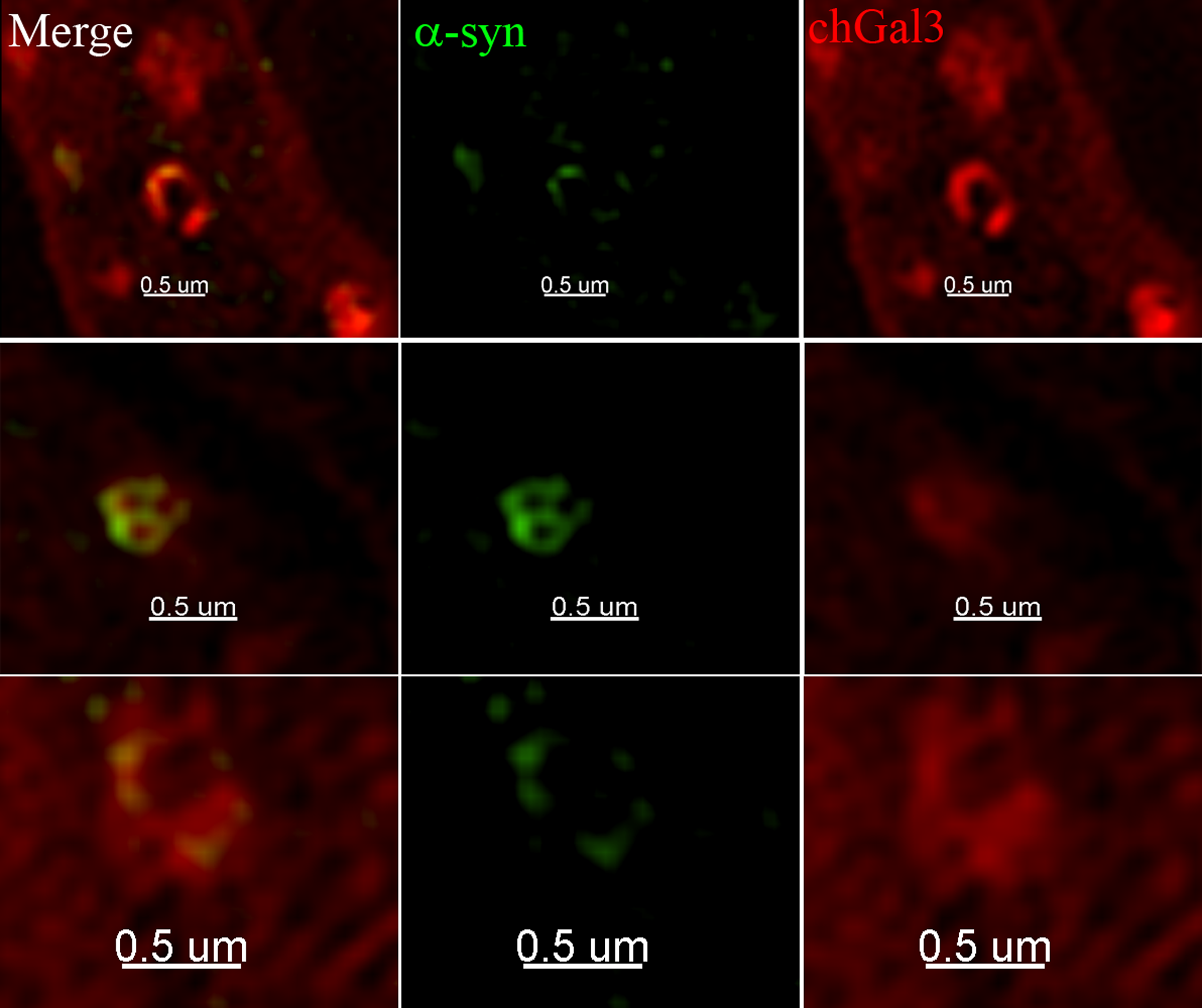

Supplement: Figure S2 — Intravesicular localization of α-synuclein. N27chGal3 cells were treated with Dylight 488 conjugated α-synuclein aggregates for 48 hours as described in the text. Shown are ruptured vesicles containing α-synuclein revealing the localization of α-synuclein to the vesicle periphery. (TIF) [file pone.0062143.s002.tif]

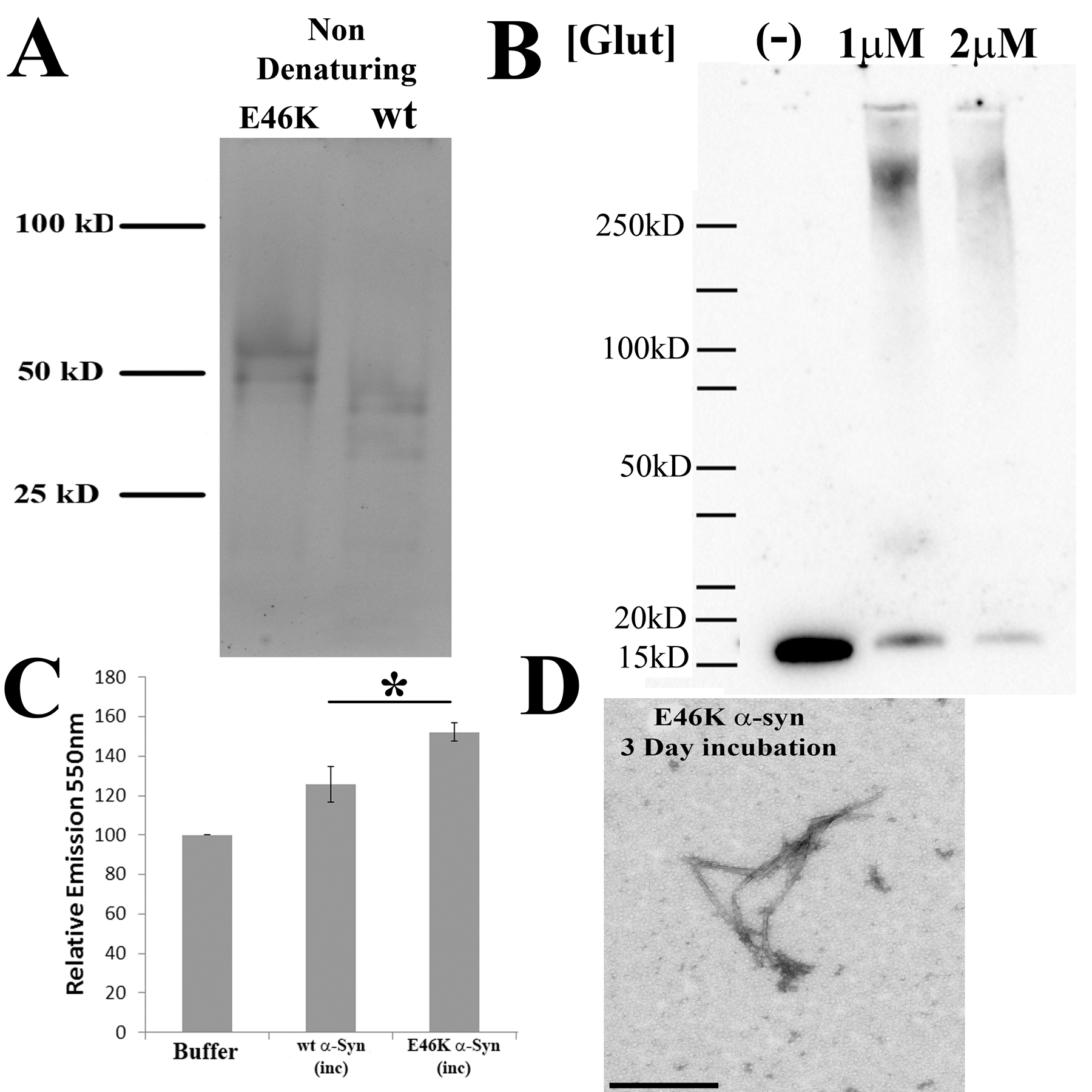

Supplement: Figure S3 — E46K aggregate characterization. E46K mutant α-synuclein was generated using in-vitro purified protein. Recombinant lyophilized E46K α-synuclein was resuspended and constantly agitated for three days at 37°C. A. The aggregates generated in this fashion were run on a non-denaturing gel, fixed and stained with Coomassie brilliant blue. E46K α-synuclein ran at a higher molecular weight than the wild-type α-synuclein on the non-denaturing gel. B. Following incubation for 3 days as described, E46K α-synuclein preparations were fixed with glutaraldehyde at the indicated concentration for 15 minutes at room temperature. C. The fibrillar content of E46K aggregates was assessed using K114 staining. E46K aggregates had significantly more fibrillar content than wt aggregates following identical treatement (*P<0.01). Results are representative of at least three independent experiments D. TEM image of E46K α-synuclein fibril. (TIF) [file pone.0062143.s003.tif]
